# Aldosterone and Mortality in Hemodialysis Patients: Role of Volume Overload

**DOI:** 10.1371/journal.pone.0057511

**Published:** 2013-02-28

**Authors:** Szu-Chun Hung, Yao-Ping Lin, Hsin-Lei Huang, Hsiao-Fung Pu, Der-Cherng Tarng

**Affiliations:** 1 Division of Nephrology, Buddhist Tzu Chi General Hospital, Taipei Branch, Taipei, Taiwan; 2 Division of Nephrology, Department of Medicine, and Immunology Research Center, Taipei Veterans General Hospital, Taipei, Taiwan; 3 Department and Institute of Physiology, School of Medicine, National Yang-Ming University, Taipei, Taiwan; University of Florida, United States of America

## Abstract

**Background:**

Elevated aldosterone is associated with increased mortality in the general population. In patients on dialysis, however, the association is reversed. This paradox may be explained by volume overload, which is associated with lower aldosterone and higher mortality.

**Methods:**

We evaluated the relationship between aldosterone and outcomes in a prospective cohort of 328 hemodialysis patients stratified by the presence or absence of volume overload (defined as extracellular water/total body water >48%, as measured with bioimpedance). Baseline plasma aldosterone was measured before dialysis and categorized as low (<140 pg/mL), middle (140 to 280 pg/mL) and high (>280 pg/mL).

**Results:**

Overall, 36% (n = 119) of the hemodialysis patients had evidence of volume overload. Baseline aldosterone was significantly lower in the presence of volume overload than in its absence. During a median follow-up of 54 months, 83 deaths and 70 cardiovascular events occurred. Cox multivariate analysis showed that by using the low aldosterone as the reference, high aldosterone was inversely associated with decreased hazard ratios for mortality (0.49; 95% confidence interval, 0.25–0.76) and first cardiovascular event (0.70; 95% confidence interval, 0.33−0.78) in the presence of volume overload. In contrast, high aldosterone was associated with an increased risk for mortality (1.97; 95% confidence interval, 1.69–3.75) and first cardiovascular event (2.01; 95% confidence interval, 1.28−4.15) in the absence of volume overload.

**Conclusions:**

The inverse association of aldosterone with adverse outcomes in hemodialysis patients is due to the confounding effect of volume overload. These findings support treatment of hyperaldosteronemia in hemodialysis patients who have achieved strict volume control.

## Introduction

Cardiovascular disease (CVD) is the leading cause of death in patients with end-stage renal disease (ESRD) [1]. There is accumulating evidence that aldosterone, in addition to its classical role in regulating fluid and electrolyte balance, plays a significant role in the pathogenesis of CVD [2]. Patients with chronic kidney disease (CKD) have higher aldosterone concentrations than the general population [3], suggesting that aldosterone might modulate the development of CVD in CKD. However, higher aldosterone levels are associated with lower mortality in CKD patients on hemodialysis [4], [5], which is in marked contrast to findings from prospective studies in the general population and in early CKD [6], [7]. A similar inverse association of serum cholesterol levels with mortality has been previously documented in dialysis patients [8]–[10]. It has been suggested that this paradoxical association results from a confounding effect of inflammation and/or malnutrition, which leads to lower cholesterol levels and higher mortality (the so called reverse epidemiology).

Volume overload is a common finding in dialysis patients and has been recognized as an important contributor to an adverse prognosis [11], [12]. This factor may explain the inverse association between aldosterone level and mortality because volume overload is strongly associated with lower aldosterone levels and higher mortality [13]. In view of the particularly high incidence of CVD in dialysis patients, a better understanding of the diagnostic implications of aldosterone levels in these patients is needed. Therefore, we investigated whether the association between aldosterone levels and mortality would be modified by the presence of volume overload.

## Materials and Methods

### Ethics Statement

The study complied with the Declaration of Helsinki and was approved by the institutional review board of National Yang-Ming University Hospital. All participants gave their written informed consent before inclusion.

### Patient Population

This prospective cohort study was conducted at the dialysis centers of affiliated hospitals of National Yang-Ming University, Taipei. The study subjects were recruited from November 1 to December 31, 2004. Initially, all patients (n = 418) undergoing hemodialysis were screened, and 366 clinically stable patients aged older than 20 years, who had been on hemodialysis for more than 6 months, were included. Exclusion criteria were dialysis for less than 12 h per week; inadequacy of dialysis, defined as Kt/V urea <1.2; conditions of malignancy, infectious disease, sepsis, or hepatobiliary disease; and unwillingness to participate in this study. Finally, the study population of 328 patients (188 men and 140 women; mean age of 59 years) was followed up until June 30, 2009. All the patients were subjected to a standard bicarbonate dialysis session with use of 137 mEq/L sodium and 2.0 mEq/L potassium dialysate. Hemodialysis was performed three times weekly using single-use dialyzers with a membrane surface area of 1.6–1.7 m^2^.

### Laboratory Investigations

Blood samples were drawn from patients who had fasted overnight before the start of a mid-week dialysis session, and heparin was then administered. Plasma and serum were separated and kept frozen at –70°C when not analyzed immediately. Plasma aldosterone levels were measured according to the manufacturer’s instructions using a commercially available radioimmunoassay kit (Diagnostic Systems Laboratories, Webster, TX). The intra- and inter-assay coefficients of variation were 3.4% and 8.9%, respectively, at an aldosterone level of 60 pg/mL, 4.7% and 7.6%, respectively, at a level of 250 pg/mL, and 4.0% and 5.2%, respectively, at a level of 500 pg/mL. Albumin, urea, creatinine, calcium, phosphate, iron, and total iron-binding capacity (TIBC) in serum were determined with a Hitachi 7600 autoanalyzer (Roche Modular; Hitachi Ltd, Tokyo, Japan) using commercial kits. Serum high-sensitivity C-reactive protein (hs-CRP) levels were measured using an immunoturbidimetric assay and rate nephelometry (IMMAGE; Beckman Coulter, Galway, Ireland). The adequacy of dialysis was estimated by measuring mid-week urea clearance (Kt/V urea) using the standard method [14]. Blood pressure (BP) was measured and recorded by an automated sphygmomanometer. Pre-dialysis BP (before placement of a dialysis needle) was measured in the nonaccess arm after a 5-minute rest while the patient was seated with both feet on the floor.

### Bioimpedance Study

Multifrequency bioimpedance method (Model 310 Bioimpedance Analyzer; Biodynamics, Seattle, WA) was performed within 30 minutes after a dialysis session at presumed dry weight. The ratio of extracellular water to total body water (ECW/TBW) was then taken as a measure of volume status. To avoid inter-observer variation, a single well-trained dietitian was involved in the measurement of bioimpedance.

### Outcomes

In all patients, a thorough medical history was taken at the time of study enrollment. Presence of CVD was defined as a medical history and clinical findings of congestive heart failure, coronary artery disease, cerebrovascular disease, and/or peripheral vascular disease. No major modifications were made in dialysis treatments during the follow-up period. The primary outcome measures were death from any cause and CV events from the time of inclusion in the study. CV events included fatal and nonfatal myocardial infarction, stroke and congestive heart failure, as well as complicated peripheral vascular disease and sudden death. A trained physician who had no knowledge of the results of plasma aldosterone measurements independently reviewed all suspected CV events by examining each medical chart.

### Statistical Analysis

**Figure 1:**
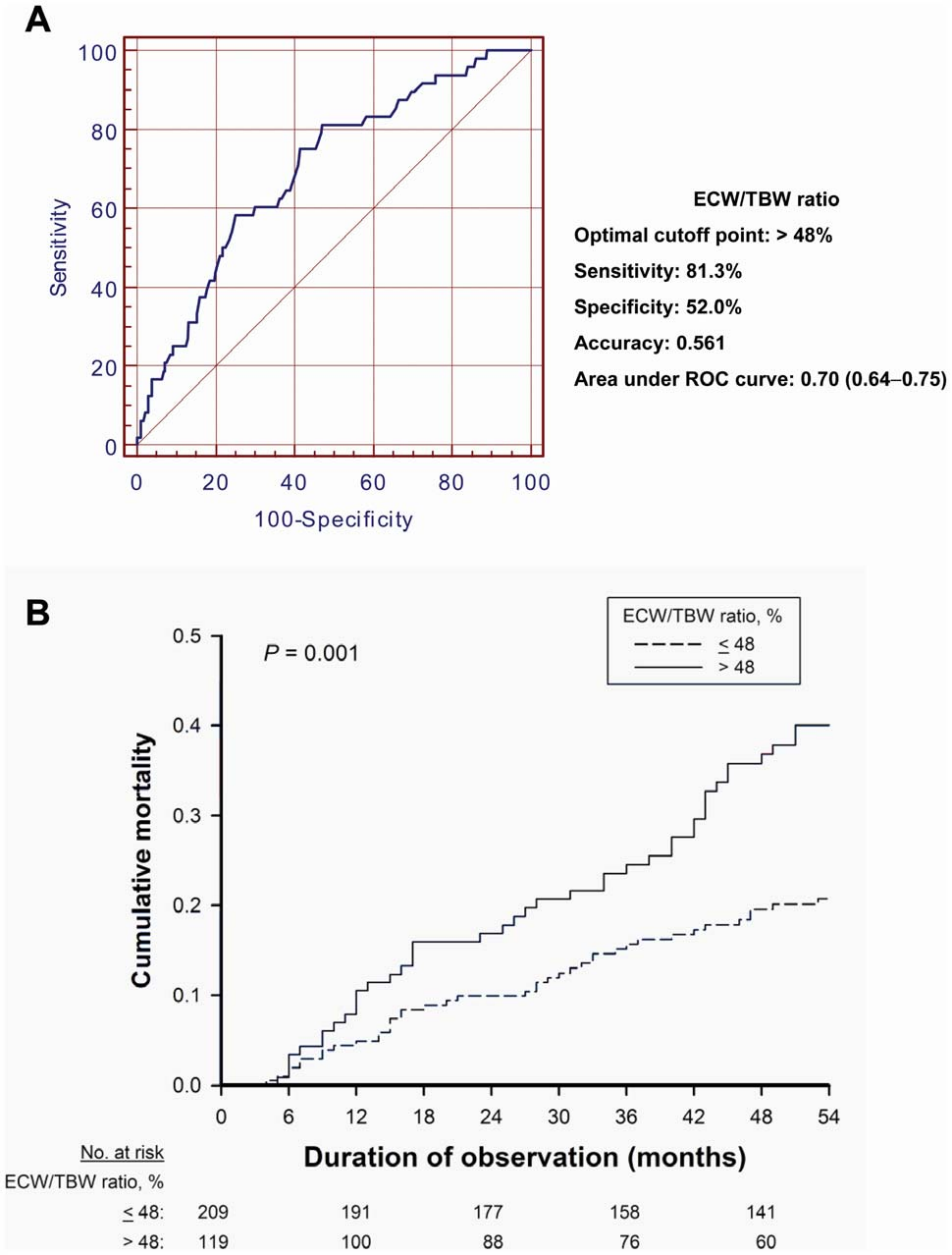
Volume status for predicting mortality. The receiver operating characteristic (ROC) curve for prediction of all-cause mortality constructed using the ratio of extracellular water to total body water (ECW/TBW) at baseline (A). The optimal cutoff point for ECW/TBW is listed in the attached table, along with the sensitivity, specificity and accuracy for predicting mortality at the end of the follow-up period. The area under ROC curve is significantly larger than 0.5. Kaplan-Meier analysis curve for all-cause mortality in relation to the ECW/TBW at baseline, stratified by the cutoff point among hemodialysis patients (B).

All variables were expressed as percentages for categorical data and as means ± SDs or medians and interquartile ranges (IQRs) for continuous data with or without a normal distribution, respectively. The baseline characteristics of the 2 study subgroups with ECW/TBW ≤48% and >48% were compared using a *t*-test, *x* ^2^ statistics, and Mann-Whitney U test as appropriate. Potential differences among the 3 patient groups for each baseline plasma aldosterone tertile were assessed by an analysis of variance (ANOVA), *x* ^2^ statistics, or the Kruskal-Wallis test, as appropriate. Receiver operating characteristic (ROC) curves were constructed for prediction of mortality using ECW/TBW at baseline. The optimal cutoff point for volume overload (ECW/TBW >48%) is listed in Figure 1, along with the sensitivity, specificity and accuracy for predicting mortality at the end of the follow-up period. Confidence intervals (CI) for the area under the ROC curves were calculated using nonparametric assumptions. Univariate correlations between ECW/TBW or plasma aldosterone and potentially explanatory variables were assessed by Pearson correlation analyses.

The Kaplan-Meier method was used to describe survival curves. Individuals were censored at the time of kidney transplantation, peritoneal dialysis, and withdrawal from the study, or at the end of the follow-up period on June 30, 2009. Cox proportional hazards models were used to investigate the role of volume overload as a potential effect modifier for relationships between diverse independent variables and either all-cause mortality or first CV event. For all multivariate survival analyses, clinically relevant variables with a P value ≤0.1 in the univariate analysis were fitted and the backward selection procedure was used for model selection [15]. The aldosterone variables were modeled as categorical variables (<140, 140–280, and >280 pg/mL) or continuous variables (per 100 pg/mL). Adjusted hazard ratios with 95% CI were reported. P values less than 0.05 were considered statistically significant. P values for interactions between the volume indicators and the aldosterone levels were computed using likelihood ratio tests. All statistical analyses were performed using the computer software Statistical Package for the Social Science, version 16.0 (SPSS Inc., Chicago, IL).

## Results

### Clinical Characteristics

The mean baseline age of the 328 patients was 59±13 years, 57.3% were male, and 29.3% had diabetes. The baseline characteristics for the patient groups divided by the absence or presence of volume overload [defined as ECW/TBW >48%] are presented in Table 1. Overall, 36% (n = 119) of the dialysis patients had evidence of volume overload. The presence of volume overload was associated with older age, diabetes, prior CVD, hypertension, higher inflammatory markers, and lower aldosterone, albumin, calcium, phosphate, and hemoglobin levels. There were no significant differences between groups regarding the type of antihypertensive medications, dialysis prescription, and serum potassium levels. The median plasma aldosterone concentration was 184 pg/mL (IQR: 97–484 pg/mL), which is above the 75^th^ percentile for aldosterone in a large cohort of non-CKD patients referred for coronary angiography [3]. Table 2 shows the baseline characteristics stratified by both volume status and plasma aldosterone categories. In patients without volume overload, hs-CRP and interleukin-6 (IL-6) levels were significantly higher in patients in the >280 pg/mL aldosterone category than in patients in the <140 pg/mL aldosterone category (P<0.05). This relationship was inversed in patients with volume overload. The number of antihypertensive medication was positively and significantly associated with the aldosterone categories for patients without volume overload, but the trend was reversed for patients with volume overload.

**Table 1:** Baseline characteristics of the study participants by categories of ECW/TBW.

Baseline values	Total patients	ECW/TBW ≤48%	ECW/TBW >48%	P*-*value^a^
	(n = 328)	(n = 209)	(n = 119)	
Age (years)	59±13	55±12	65±11	<0.001^b^
Male (%)	57.3	59.8	52.9	0.23^c^
Smoking history (%)	31.7	32.1	31.1	0.86^c^
Diabetes mellitus (%)	29.3	19.1	47.1	<0.001^c^
Prior CVD (%)	23.4	18.2	32.8	0.002^c^
Hypertension (%)	48.2	40.6	61.3	<0.001^c^
Systolic BP (mmHg)	136±24	134±22	140±26	0.04^b^
Diastolic BP (mmHg)	76±12	76±11	76±14	0.99^b^
RAAS blockade (%)	27.1	24.9	31.1	0.22^c^
Calcium channel blocker (%)	62.2	59.8	66.4	0.31^c^
β-blocker (%)	32.6	32.0	33.6	0.53^c^
No. of antihypertensives	2.0±1.3	2.0±1.1	2.1±1.5	0.72^b^
Statin use (%)	15.9	16.3	15.1	0.79^c^
HD duration (months)	66±54	67±53	65±56	0.84^b^
Kt/V urea	2.0±0.6	2.0±0.5	2.1±0.7	0.11^b^
Intradialytic UF (L)	2.9±0.6	2.9±0.7	2.8±0.5	0.87^b^
BMI (kg/m^2^)	22.6±3.5	22.0±3.1	23.3±4.1	0.02^b^
ECW (L)	13.0±2.1	12.5±2.1	13.7±2.0	<0.001^b^
TBW (L)	28.4±4.6	29.3±4.8	26.9±3.9	<0.001^b^
ECW/TBW (%)	45.8±5.0	42.9±3.4	50.9±2.8	<0.001^b^
Aldosterone (pg/mL)	184 (97–484)	215 (99–768)	166 (95–263)	0.02^d^
hs-CRP (mg/L)	3.94 (1.29–7.86)	3.74 (1.55–6.74)	4.10 (1.24–8.09)	0.04^d^
IL-6 (pg/mL)	2.92 (2.21–7.87)	2.80 (1.78–5.53)	4.59 (2.66–9.67)	<0.001^d^
Albumin (g/L)	39.3±3.5	39.9±3.2	38.2±3.7	<0.001^b^
Total cholesterol (mg/dL)	186±33	188±32	180±35	0.16^b^
Triglyceride (mg/dL)	127±61	130±64	121±57	0.18^b^
Potassium (mmol/L)	4.01±0.36	4.02±0.37	3.98±0.37	0.28^b^
Calcium (mg/dL)	9.7±0.7	9.8±0.7	9.6±0.8	0.03^b^
Phosphate (mg/dL)	5.0±1.4	5.2±1.4	4.8±1.3	0.002^b^
Hemoglobin (g/dL)	10.5±1.5	10.8±1.5	9.8±1.4	<0.001^b^
EPO dose (u/kg/wk)	66±46	55±47	87±36	<0.001^b^
Ferritin (µg/L)	344 (201–538)	291 (129–439)	361 (255–606)	0.001^d^
Transferrin saturation (%)	27±13	27±13	27±14	0.77^b^

Abbreviations: BMI denotes body mass index; BP, blood pressure; CVD, cardiovascular disease; ECW, extracellular water; ECW/TBW, ratio of extracellular water to total body water; EPO, erythropoietin; HD, hemodialysis; hs-CRP, high-sensitivity C-reactive protein; IL-6, inerleukin-6; RAAS, renin-angiotensin-aldosterone system; TBW, total body water; UF, ultrafiltration.

a Comparison between two groups of patients with ECW/TBW ≤48% and >48%.

Statistical analysis by ^b^Student *t*-test, ^c^Pearson *x* ^2^ test, and ^d^Mann-Whitney U test.

Prior CVD category consisted of congestive heart failure, coronary artery disease, cerebrovascular disease, and peripheral arterial disease.

**Table 2:** Baseline characteristics of the study participants by categories of ECW/TBW and plasma aldosterone.

	ECW/TBW ≤48%			ECW/TBW >48%				
	Plasma aldoterone, pg/mL			Plasma aldoterone, pg/mL				
	<140	140–280	>280		<140	140–280	>280	
Baseline values	(n = 69)	(n = 53)	(n = 87)		(n = 39)	(n = 56)	(n = 24)	
Age (years)	55±12	57±11	54±11		67±9	66±13	64±9	
Male (%)	63.8	54.7	59.8		51.3	53.6	54.2	
Smoking history (%)	34.8	32.1	29.9		30.8	28.6	37.5	
Diabetes mellitus (%)	23.2	24.5	12.6		38.5	51.8	50.0	
Prior CVD (%)	20.3	22.6	13.8		38.5	30.4	29.2	
Hypertension (%)	39.4	41.8	41.3		67.9	66.7	41.6b,c	
Systolic BP (mmHg)	132±19	134±24	135±24		143±27	140±25	135±26b,c	
Diastolic BP (mmHg)	76±9	76±11	75±11		74±12	77±18	76±7	
RAAS blockade (%)	15.9	28.3	29.9		28.2	30.4	37.5	
No. of antihypertensives	1.5±1.0	2.0±1.0	2.4±1.5^b^		2.5±1.5	2.4±1.5	1.4±1.0b,c	
Statin use (%)	11.6	17.0	19.5		17.9	14.3	12.5	
HD duration (months)	67±49	65±53	68±56		59±47	67±52	74±74	
Kt/V urea	2.0±0.6	2.0±0.3	2.0±0.4		2.0±0.4	2.3±0.9	2.0±0.3	
BMI (kg/m^2^)	21.8±3.1	21.7±2.8	22.6±3.1		23.1±5.2	23.5±3.4	22.5±4.8	
ECW (L)	12.4±1.5	12.3±2.2	12.8±2.2		13.9±2.2	13.0±1.9	13.6±2.1	
TBW (L)	28.2±3.7	28. ±9 4.8	30.1±5.1		27.1±4.6	26.7±3.4	27.3±4.4	
ECW/TBW (%)	44.2±2.9	42.7±4.1^b^	42.7±3.1^b^		51.4±2.9	50.9±2.8	49.9±2.0b,c	
hs-CRP (mg/L)	2.53 (1.09–4.99)	3.85 (1.26–5.79)	4.76 (1.62–7.55)^b^		6.12 (2.28–8.63)	4.70 (0.94–8.06)	1.53 (0.59–5.82)^b^	
IL-6 (pg/mL)	2.42 (1.62–4.44)	3.10 (1.87–5.84)	3.85 (2.19–6.04)^b^		5.42 (2.52–11.57)	4.86 (3.04–12.36)	3.62 (2.55–6.43)^b^	
Albumin (g/L)	39.5±3.1	39.9±3.4	40.0±3.2		36.8±1.3	38.9±3.5	39.3±4.0^b^	
Total cholesterol (mg/dL)	193±45	189±40	183±30		177±32	180±34	185±38	
Triglyceride (mg/dL)	133±68	130±60	128±48		122±53	120±47	121±49	
Potassium (mmol/L)	4.03±0.37	4.02±0.37	4.01±0.37		4.00±0.36	3.99±0.37	3.98±0.37	
Calcium (mg/dL)	9.8±0.7	9.6±0.7	9.8±0.7		9.4±0.9	9.7±0.8	9.4±0.6	
Phosphate (mg/dL)	4.8±1.2	5.1±1.4	5.5±1.5		4.5±1.2	4.8±1.3	4.8±1.2	
Hemoglobin (g/dL)	10.8±1.2	10.6±1.6	10.8±1.6		9.7±1.3	9.8±1.4	10.0±1.2	
EPO dose (u/kg/wk)	51±44	72±50^b^	52±45^c^		89±31	87±41	77±36	
Ferritin (µg/L)	323 (157–407)	277 (142–541)	271 (101–466)		370 (272–675)	393 (264–593)	278 (239–514)	
Transferrin saturation (%)	29±12	24±10	28±14		27±14	27±14	26±15	

For abbreviations see Table 1.

a Comparisons among three groups in patients with ECW/TBW ≤48% and >48%, respectively. Statistical analysis by one-way ANOVA test, Pearson *x* ^2^ test, and Kruskal-Wallis test as appropriate.

b P<0.05 versus patients with plasma aldosterone of <140 pg/mL.

c P<0.05 versus patients with plasma aldosterone of 140–280 pg/mL.

Prior CVD category consisted of congestive heart failure, coronary artery disease, cerebrovascular disease, and peripheral arterial disease.

Table 3 shows univariate correlations between ECW/TBW or plasma aldosterone levels and potentially explanatory variables. The ECW/TBW was negatively correlated with plasma aldosterone, hemoglobin, albumin, calcium, and phosphate levels and was positively correlated with age, hs-CRP, IL-6, ferritin levels, and systolic BP. In contrast, plasma aldosterone was positively associated with albumin and phosphate levels and was negatively associated with age, ECW/TBW, ferritin and systolic BP.

**Table 3:** Univariate correlations between ECW/TBW or plasma aldosterone and potentially explanatory variables.

	ECW/TBW		Aldosterone	
Variables	*r*	P-value	*r*	P-value
Age (years)	−0.555	<0.001	−0.286	<0.001
Body mass index (kg/m^2^)	−0.136	0.02	−0.038	0.54
ECW/TBW (%)	–	–	−0.200	0.001
Aldosterone (pg/mL)	−0.200	0.001	–	–
hs-CRP (mg/L)	−0.180	0.002	−0.013	0.81
IL-6 (pg/mL)	−0.310	<0.001	−0.069	0.22
Hemoglobin (g/dL)	−0.368	<0.001	−0.067	0.24
Serum ferritin (µg/L)	−0.208	<0.001	−0.169	0.003
Transferrin saturation (%)	−0.092	0.10	−0.029	0.61
Albumin (g/L)	−0.337	<0.001	−0.189	0.001
Calcium (mg/dL)	−0.123	0.03	−0.082	0.15
Phosphate (mg/dL)	−0.177	0.001	−0.222	<0.001
Potassium (mmol/L)	−0.080	0.19	−0.024	0.69
Systolic BP (mmHg)	−0.123	0.03	−0.135	0.02
Diastolic BP (mmHg)	−0.032	0.57	−0.081	0.16
Kt/V urea	−0.089	0.06	−0.028	0.63

For abbreviations see Table 1.

### Follow-up Data

**Figure 2:**
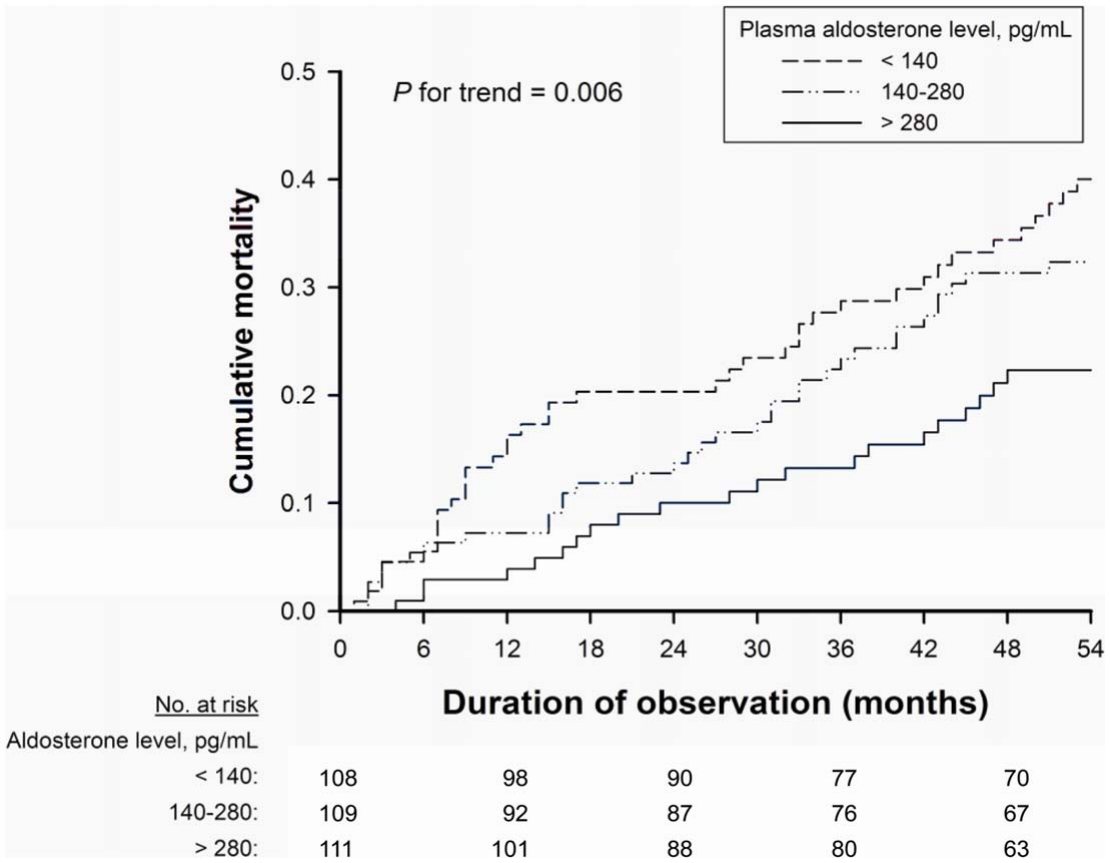
Kaplan-Meier mortality curves according to aldosterone tertile. All-cause mortality in relation to plasma aldosterone levels at baseline among hemodialysis patients.

**Figure 3:**
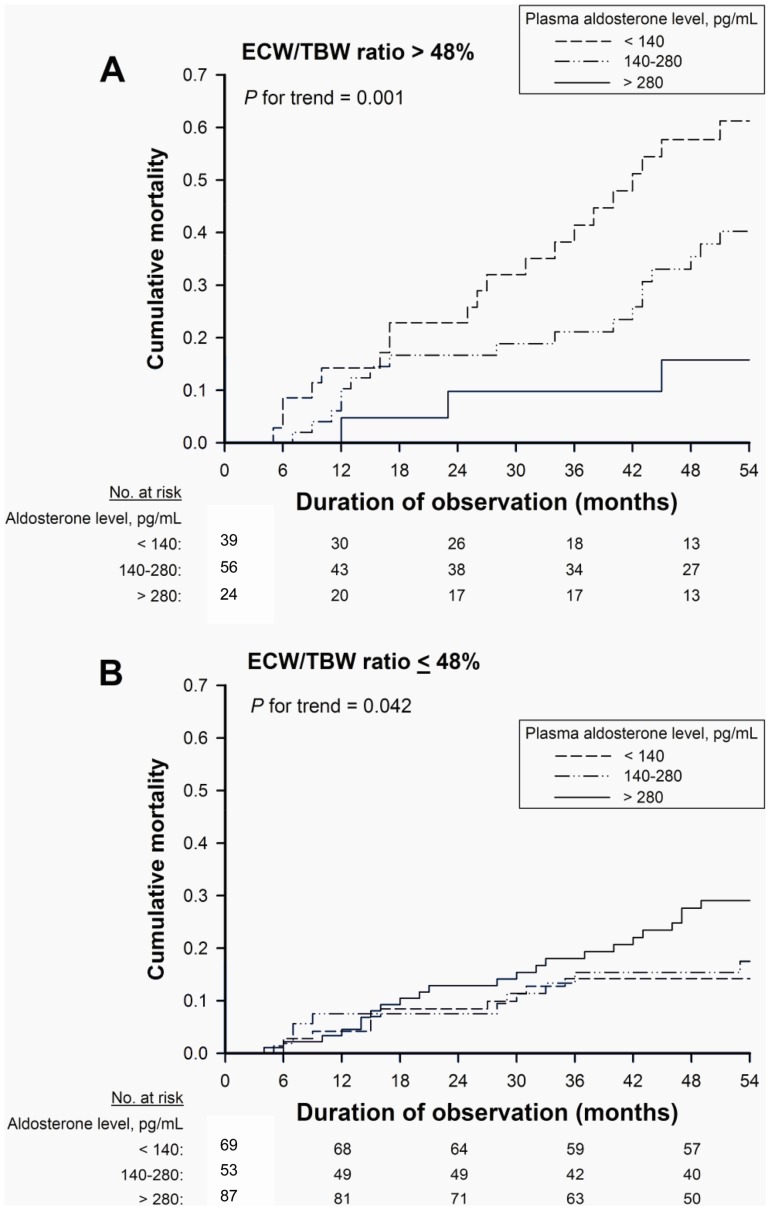
Kaplan-Meier mortality curves according to aldosterone tertile, modified by volume status. All-cause mortality in relation to plasma aldosterone levels at baseline modified by the ratio of extracellular water to total body water (ECW/TBW) >48% (A) and ≤48% (B) among hemodialysis patients.

During the follow-up period, 16 patients received kidney transplants, and 4 patients transitioned to peritoneal dialysis. Thirty-three patients who were transferred to other dialysis units were followed up using questionnaire forms completed by the attending physicians at the units. At the end of the follow-up period, 225 patients were confirmed to be alive on hemodialysis treatment, and 83 patients died while being treated; 37 (44.6%) of these deaths were due to CVD-related causes. There were 70 CV events in the median follow-up period of 54 months (IQR: 27–107 months). In unadjusted analysis, lower aldosterone levels were associated with higher mortality in the overall cohort (P for trend = 0.006) (Fig. 2) and in the presence of volume overload (P for trend = 0.001) (Fig. 3A), respectively. However, in the absence of volume overload, the association was reversed, with higher aldosterone levels associated with higher mortality (P for trend = 0.042) (Fig. 3B).

Multivariate analysis with plasma aldosterone, age, gender, prior CVD, presence of diabetes mellitus, smoking status, body mass index, systolic blood pressure, dialysis vintage, baseline levels of serum albumin, total cholesterol, hemoglobin, ferritin, calcium×phosphate, Kt/V urea, and IL-6 as the independent variables demonstrated that plasma aldosterone, age, prior CVD, serum albumin, and IL-6 were independently related to mortality and first CV event. The inverse association of aldosterone level with mortality, both in the entire patient population and in the presence of volume overload (ECW/TBW >48%), was also seen in adjusted analysis (Table 4). Furthermore, volume overload was found to modify the risk relationship between aldosterone and first CV event in the Cox regressions with plasma aldosterone level as a categorical (interaction, P = 0.02) or a continuous measure (interaction, P = 0.03). In the absence of volume overload (ECW/TBW ≤48%), the aldosterone category was positively associated with all-cause mortality and first CV event. Analyzing aldosterone as a continuous variable also showed the similar association of aldosterone with all-cause mortality and first CV event.

**Table 4:** Hazard ratios for mortality and first cardiovascular event by categorical or continuous measure of plasma aldosterone modified by ECW/TBW.

	Overall	ECW/TBW >48%	ECW/TBW ≤48%	P-value^a^
	(n = 328)	(n = 119)	(n = 209)	
**All-cause Mortality, Adjusted HR (95% Confidence Interval)**				
**Categorical measure**				
Aldosterone <140 pg/mL	1.00	1.00	1.00	0.01
Aldosterone 140–280 pg/mL	0.87 (0.58−1.31)	0.82 (0.43−1.45)	1.15 (0.91−2.23)	
Aldosterone >280 pg/mL	0.65 (0.48–0.91)	0.49 (0.25−0.76)	1.97 (1.69−3.75)	
Age, per 1 year	1.05 (1.03−1.07)	1.03 (1.00−1.08)	1.07 (1.02−1.12)	
Prior CVD	2.54 (1.54−4.17)	5.29 (2.44−11.49)	2.35 (1.87−6.36)	
Albumin, per 1 g/L	0.92 (0.85−0.98)	0.87 (0.75−0.91)	0.91 (0.79−0.92)	
IL-6, per 1 pg/mL	1.07 (1.01−1.14)	1.11 (1.01−1.23)	1.08 (1.04−1.12)	
**Continuous measure**				
Aldosterone, per 100 pg/mL	0.75 (0.60−0.91)	0.84 (0.67−0.96)	1.61 (1.20−2.73)	0.03
Age, per 1 year	1.05 (1.03−1.08)	1.03 (0.99−1.07)	1.07 (1.02−1.12)	
Prior CVD	2.58 (1.40−4.76)	5.52 (2.53−12.07)	2.14 (1.80−5.67)	
Albumin, per 1 g/L	0.92 (0.85−0.99)	0.85 (0.77−0.94)	0.93 (0.89−0.97)	
IL-6, per 1 pg/mL	1.05 (1.02−1.13)	1.11 (1.02−1.27)	1.07 (1.03−1.11)	
**First CV Event, Adjusted HR (95% Confidence Interval)**				
**Categorical measure**				
Aldosterone <140 pg/mL	1.00	1.00	1.00	0.02
Aldosterone140–280 pg/mL	0.89 (0.65−1.41)	0.81 (0.51−1.89)	1.21 (0.92−2.43)	
Aldosterone >280 pg/mL	0.79 (0.41−0.81)	0.70 (0.33−0.78)	2.01 (1.28−4.15)	
Age, per 1 year	1.03 (1.02−1.06)	1.02 (0.97−1.08)	1.04 (0.99−1.10)	
Prior CVD	5.08 (2.57−10.09)	6.45 (2.52−16.52)	3.41 (1.24−9.39)	
Albumin, per 1 g/L	0.89 (0.80−0.99)	0.80 (0.65−0.98)	0.82 (0.68−0.98)	
IL-6, per 1 pg/mL	1.14 (1.04−1.25)	1.14 (1.02−1.29)	1.21 (1.01−1.45)	
**Continuous measure**				
Aldosterone, per 100 pg/mL	0.80 (0.69−0.95)	0.69 (0.57−0.90)	1.67 (1.12−2.89)	0.03
Age, per 1 year	1.04 (1.01−1.08)	1.03 (0.97−1.09)	1.05 (0.99−1.12)	
Prior CVD	5.10 (2.26−11.56)	6.45 (2.52−16.52)	3.42 (1.24−9.39)	
Albumin, per 1 g/L	0.89 (0.79−0.96)	0.81 (0.65−0.99)	0.82 (0.68−0.98)	
IL-6, per 1 pg/mL	1.13 (1.02−1.25)	1.15 (1.02−1.29)	1.21 (1.01−1.45)	

Abbreviations: CI denotes confidence interval; CV, cardiovascular; CVD, cardiovascular disease; ECW/TBW, ratio of extracellular water to total body water; HR, hazard ratio; IL-6, inerleukin-6.

a The interaction P values assessed the modifying effect of volume overload (ECW/TBW >48%) on the risk relationship between plasma aldosterone and overall mortality and CV events.

## Discussion

This study demonstrates an inverse association of aldosterone levels with all-cause mortality and CV event rates in the presence of volume overload. This represents a paradoxical effect of volume status on mortality. In contrast, there was a significant, graded, and positive association of aldosterone levels with all-cause mortality and CV event rates in the 64% of participants without volume overload. Accordingly, some ESRD patients with low aldosterone levels have a low risk of adverse outcomes, as in the general population, whereas others have a high risk because they are in a state of volume overload, which lowers aldosterone levels and increases the risk of mortality and CV events. These findings underline the importance of hyperaldosteronemia as a risk factor for adverse long-term outcomes among patients with ESRD, and of the masking of this association among individuals with volume overload.

A growing body of evidence has linked aldosterone excess to the development and progression of different CVD processes, including hypertension, congestive heart failure, and coronary artery disease [16], [17]. Plasma aldosterone levels are markedly elevated among patients with CKD [3]–[5] which is similar to the remnant kidney model in the rat [18]. In a study of 28 selected patients with varying degrees of CKD and normal serum potassium levels and plasma renin activity, Hene et al. showed that aldosterone levels were elevated when creatinine clearance was less than 50% of normal, increasing three- to four-fold above normal levels as clearance values decreased [3]. Therefore, aldosterone-mediated CV damage might be amplified by decreased kidney function, which by itself is a potent CV risk factor [7].

Plasma aldosterone levels are influenced by a number of factors, including potassium and volume status. Our results showed that a relatively lower aldosterone level was in fact a surrogate marker of volume overload in hemodialysis patients. In healthy volunteers with salt loading and in hemodialysis patients with increased inter-dialytic weight gain, expansions of ECW led to reciprocal declines in plasma aldosterone concentrations. The relationship was more profound in healthy volunteers than in hemodialysis patients. As a result, the shift of the volume-aldosterone curve in hemodialysis patients suggests that ESRD is a state of high volume and inappropriately high aldosterone levels [19]. ESRD patients are particularly vulnerable to sudden cardiac death resulting from left ventricular hypertrophy (LVH) and fibrosis [20]. Cumulative evidence has indicated that aldosterone, beyond its classical actions on epithelial cells with BP-dependent organ damage, exerts non-classical effects on interstitial tissues, which are involved in cardiac and renal fibrosis [21]–[24]. These non-epithelial effects of aldosterone are exaggerated in conditions of elevated aldosterone levels and expanded ECW, such as ESRD [25].

In a large cohort of non-CKD patients scheduled for coronary angiography, variations in aldosterone concentrations within the normal range were associated with increased all-cause and CV mortality independent of major established CV risk factors [6]. In the same cohort, the association of higher plasma aldosterone concentrations with overall CV mortality and sudden cardiac death was stronger for patients with decreased kidney function [7]. Furthermore, a recent study by Edwards et al. also showed that the use of spironolactone reduced left ventricular (LV) mass and improved arterial stiffness in early-stage CKD [26]. Unfortunately, the largest trials to date that used mineralocorticoid receptor antagonists and demonstrated a mortality benefit in patients with severe heart failure due to systolic LV dysfunction or acute myocardial infarction [27], [28], excluded patients with moderate to advanced CKD. Once CKD patients have progressed to ESRD, aldosterone levels may remain elevated [29]–[31]. The markedly elevated levels of aldosterone levels seen in ESRD suggest that mineralocorticoid receptor blockade could emerge as a crucial strategy against CVD in this population [32]. Randomized controlled trials are required to confirm our preliminary findings and define the risk of potential hazards, particularly those involving hyperkalemia [33].

The present study has a number of limitations. First, the modifying effect of volume overload on the risk relationship between aldosterone and mortality may still be subject to residual confounding. Furthermore, as with any cohort study, this study cannot establish causality between volume overload or aldosterone levels and mortality, and we caution against translating the results of observational studies into therapeutic practice. Finally, the impact of volume overload on the association between aldosterone and mortality might be different at different stages of CKD. It is unclear whether findings from our study of patients on dialysis can be extrapolated to patients who have moderate to advanced CKD and are not yet on dialysis.

Our study has biological plausibility and important therapeutic implications. In CKD patients, a progressive decline in glomerular filtration rate, activation of the renin-angiotensin-aldosterone system (RAAS), and superimposed CV comorbidities contribute to salt and water retention. Volume overload and inappropriately high aldosterone levels in CKD patients resulted in more severe LVH and increased arterial stiffness [34], [35], both of which are independent predictors of CV mortality in dialysis patients [36], [37]. Achieving strict volume control appears to be an imperative therapeutic strategy for inducing regression of LVH and arterial compliance, and lowering the CVD risk in dialysis patients. However, our study provides strong evidence that favorable effects of volume correction might be negated by the simultaneous stimulation of RAAS and thus may become apparent only if this response is inhibited [38], [39].

In summary, aldosterone level is inversely associated with adverse outcomes in hemodialysis patients. Volume overload underlies this paradox. In the absence of volume overload, aldosterone is an independent risk factor for all-cause mortality and CV events in this population. These data provide evidence for the confounding and effect modification of the association of aldosterone with adverse outcomes by volume overload. Hence, further research is warranted to clarify the pathophysiological mechanisms that link volume overload and hyperaldosteronemia to increased mortality and CV events and whether therapeutic interventions to mitigate volume overload and lower aldosterone concentrations may lead to improved outcomes in dialysis patients.
